# Plasma club cell secretory protein reflects early lung injury: comprehensive epidemiological evidence

**DOI:** 10.1265/ehpm.24-00335

**Published:** 2025-04-15

**Authors:** Jiajun Wei, Jinyu Wu, Hongyue Kong, Liuquan Jiang, Yong Wang, Ying Guo, Quan Feng, Jisheng Nie, Yiwei Shi, Xinri Zhang, Xiaomei Kong, Xiao Yu, Gaisheng Liu, Fan Yang, Jun Dong, Jin Yang

**Affiliations:** 1 MOE Key Laboratory of Coal Environmental Pathogenicity and Prevention, NHC Key Laboratory of Pneumoconiosis, Department of Occupational Health, School of Public Health, Shanxi Medical University, Shanxi Key Laboratory of Environmental Health Impairment and Prevention, Xinjiannan Road 56, Taiyuan City (030001), Shanxi Province, China; 2 Xishan Coal Electricity Corporation Occupational Disease Prevention and Control Institute, Taiyuan City (030053), Shanxi Province, China; 3 NHC Key Laboratory of Pneumoconiosis, Shanxi Key Laboratory of Respiratory Diseases, Department of Pulmonary and Critical Care Medicine, The First Hospital of Shanxi Medical University, Shanxi Medical University, Jiefangnan Road 85, Taiyuan City (030001), Shanxi Province, China

**Keywords:** Coal workers’ pneumoconiosis, Cumulative respiratory dust exposure, Club cell secretory protein, Lung function

## Abstract

**Background:**

It is inaccurate to reflect the level of dust exposure through working years. Furthermore, identifying a predictive indicator for lung function decline is significant for coal miners. The study aimed to explored whether club cell secretory protein (CC16) levels can reflect early lung function changes.

**Methods:**

The cumulative respiratory dust exposure (CDE) levels of 1,461 coal miners were retrospectively assessed by constructed a job-exposure matrix to replace working years. Important factors affecting lung function and CC16 were selected by establishing random forest models. Subsequently, the potential of CC16 to reflect lung injury was explored from multiple perspectives. First, restricted cubic spline (RCS) models were used to compare the trends of changes in lung function indicators and plasma CC16 levels after dust exposure. Then mediating analysis was performed to investigate the role of CC16 in the association between dust exposure and lung function decline. Finally, the association between baseline CC16 levels and follow-up lung function was explored.

**Results:**

The median CDE were 35.13 mg/m^3^-years. RCS models revealed a rapid decline in forced vital capacity (FVC), forced expiratory volume in the first second (FEV_1_), and their percentages of predicted values when CDE exceeded 25 mg/m^3^-years. The dust exposure level (<5 mg/m^3^-years) causing significant changes in CC16 was much lower than the level (25 mg/m^3^-years) that caused changes in lung function indicators. CC16 mediated 11.1% to 26.0% of dust-related lung function decline. Additionally, workers with low baseline CC16 levels experienced greater reductions in lung function in the future.

**Conclusions:**

CC16 levels are more sensitive than lung indicators in reflecting early lung function injury and plays mediating role in lung function decline induced by dust exposure. Low baseline CC16 levels predict poor future lung function.

**Supplementary information:**

The online version contains supplementary material available at https://doi.org/10.1265/ehpm.24-00335.

## Graphical Abstract

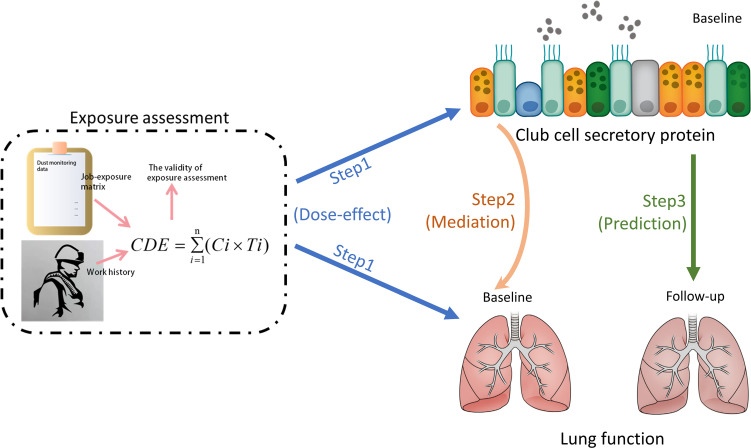

## Introduction

China’s energy structure has historically been dominated by coal and the number of new coal workers’ pneumoconiosis (CWP) cases in mainland China ranked the highest worldwide [1]. According to data released by the National Health Commission of the People’s Republic of China, the cumulative number of reported pneumoconiosis cases nationwide amounted to 306,624 from 2007 to 2022, constituting over 80 percent of the total number of occupational diseases. Furthermore, CWP accounted for more than half of the newly reported cases of pneumoconiosis. Coal is expected to account for more than 50% of China’s energy consumption in the coming years [2]. Therefore, a significant portion of the occupational population may continue to be exposed to coal mine dust, which could lead to future diagnoses of CWP. Diagnosis of pneumoconiosis is strict and requires complex evaluation procedures. The condition is often serious by the time a coal miner is diagnosed with pneumoconiosis [3]. These conditions underscore the significance of identifying feasible early biomarkers for monitoring lung function in coal miners.

In contrast to cytokines such as TNF-alpha and IL-6, which can be secreted by various cells in multiple parts of the body, club cell secretory protein (CC16) is a homodimeric protein primarily produced by the distal airway club cells [4]. Club cells are present throughout the respiratory tract epithelium from the nose to the respiratory bronchioles [5]. When the integrity of the lung blood-air barrier is compromised, it results in changes in blood concentrations of CC16. CC16 is considered a biological marker of lung epithelial injury and lung permeability, and it has anti-inflammatory properties and results in protective effects against oxidative stress in the respiratory tract [6]. The mechanism underlying pneumoconiosis remains unclear, but some studies have suggested immune dysfunction and the combined action of multiple cytokines as the main causes [7, 8]. Many epidemiological studies have shown that long-term exposure to pollutants is associated with decreased circulating CC16 levels [9, 10]. Considering the source of CC16 and its anti-inflammatory effects, CC16 may reflect early lung injury and has the potential to become a biomarker for early lung injury in coal miners. However, it still lacked epidemiological evidence and the potential of CC16 has not been thoroughly studied. Currently, the most common used research method for assessing coal miners’ dust exposure levels is solely based on working years, which may be inaccurate owing to significant variations in dust concentrations across different types of jobs [11, 12]. Nevertheless, dust exposure remains the most direct factor affecting lung function in coal miners.

Therefore, we constructed a job-exposure matrix using dust monitoring data from 2004–2021 and then retrospectively assessed the dust exposure levels in 1,461 underground workers to replace working years. Based on this assessment, the potential of CC16 to reflect early lung function injury in coal miners was explored from different aspects. The study compared whether changes in CC16 were more sensitive than lung function indicators, particularly under low dust exposure among coal miners. It also examined the role of CC16 in the decline of lung function among coal miners and evaluated its predictive ability for lung function deterioration.

## Materials and methods

### Study design and population

This cohort study started in 2021 on two coal mines that were owned by the same company and located in the same vicinity (<50 km). At baseline, we developed a standardized questionnaire to collect demographic information, including age, sex, education level, drinking and smoking status, and work history, which was administered by specially trained investigators during face-to-face interviews. Blood samples (7 mL) were collected from the participants after the provision of written informed consent. Basic information and biological samples were collected from 1,607 workers. A total of 1,461 coal miners were included in the study at baseline after excluding workers with incomplete basic information (n = 53), incomplete lung function indices (n = 13), and non-underground workers (n = 80). After more than a year, 1,268 workers were followed through the occupational health examination system because 193 participants resigned or were transferred to the surface. The process of coal miners recruitment was provided in Fig. S1. This study was approved by the Ethics Committee of the First Hospital of Shanxi Medical University (approval number K-K104).

### Occupational exposure assessment

Data on the time-weighted average concentration (TWA) of respiratory dust were collected after 2004 through personal sampling. A job-exposure matrix was constructed by collecting the dust exposure information for different types of jobs [13]. The work history of each coal miner was obtained from employment records. Missing dust exposure information was estimated using monitoring data for similar jobs or the same job at different times. The estimated cumulative respirable dust exposure for each individual was calculated by linking the job-exposure matrix with work histories as follows:

$$\mathrm{CDE}=\sum_{\text{i}=1}^{n}(C_{i}\times T_{i})$$

in which CDE represents the cumulative respiratory dust exposure, n represents the total number of job titles held by the individual throughout their work history, C_i_ represents the 8-hour time-weighted mean concentration for the i^th^ job title, and T_i_ represents the duration of employment in years for the i^th^ job.

### Lung function test

Spirometry was performed by professionally trained nurses using a lung function analyzer (CHESTGRAPH HI-101, Japan) based on the spirometry guidelines issued jointly by the American Thoracic Society (ATS) and the European Respiratory Society (ERS) [14]. Lung indicators included forced vital capacity (FVC), forced expiratory volume in one second (FEV_1_) and the ratio of the two (FEV_1_/FVC). To exclude the effects of age and height on lung function, the percentage of predicted forced vital capacity (ppFVC) and the percentage of predicted forced expiratory volume in one second (ppFEV_1_) (measured/predicted value) were also analyzed and automatically calculated using the built-in formula. The instrument was calibrated prior to each use, and the maximum value obtained from the three tests was regarded as the definitive lung function measurement.

### Plasma CC16 determination

Plasma CC16 concentrations were determined using enzyme-linked immunosorbent assay kits (R&D Systems, Minnesota, USA). The assay range was 0.8–50 ng/mL, in accordance with the manufacturer’s instructions. Optical density was converted to protein concentration by applying four-parameter logistic regression to the standard curve. All samples were tested three times. Ten randomly selected plasma samples were thoroughly mixed and used as the standards. The intra- and inter-assay coefficients of variation were 3.1% and 6.1%, respectively. After all the samples were detected, we randomly selected 10% of the samples for redetection and calculated the intraclass correlation coefficient (ICC = 0.966, *P* < 0.01).

### Definitions

Current drinkers were defined as individuals who currently consumed alcohol at least once a week for 6 months consecutively or longer. Former drinkers were defined as individuals who stopped drinking for longer than one year, whereas all others were defined as non-drinkers. The amount of smoking was calculated by multiplying the number of packs smoked per day by the duration of smoking. Regular exercise was defined as engaging in physical activity for at least three times per week, with each session lasting more than 30 minutes. Occasional exercise was defined as engaging in exercise without meeting the criteria for regular exercise, whereas all others were defined as non-exercisers. People with abnormal fasting plasma glucose (FPG) were defined as individuals with FPG levels ≥6.1 mmol/L or who take hypoglycemic medication regularly or self-reported physician-diagnosed diabetes [15]. Overweight or obesity is defined as a BMI over 24 kg/m^2^ [16]. Dysarteriotony was defined as systolic blood pressure (SBP) ≥140 mmHg or diastolic blood pressure (DBP) ≥90 mmHg for a single blood pressure measurement [17]. Restrictive ventilatory dysfunction was defined as ppFVC <80% and obstructive ventilatory dysfunction was defined as FEV_1_/FVC <70%. Workers with both types of ventilatory dysfunction were defined as mixed ventilatory dysfunction [18].

### Statistical analysis

The CDE was lg-transformed owing to skewed distribution. Coal miners were divided into two subgroups according to the median cumulative dust exposure, and their basic characteristics were described using frequency (proportion) and median (first quartile, last quartile). The Chi-square test and Kruskal–Wallis H-test were used to test for differences in categorical and numerical variables, respectively. The missing data for covariates (<5%) was estimated using multiple interpolation based on the R package ‘mice’ [19]. We established RF models to screen variables that had greater impacts on lung function and CC16 using the R package ‘randomForest’ [20]. Generalized linear regression models and RCS models were employed to investigate the dose-effect association between dust exposure and lung function and plasma CC16 concentrations.

To continue explore the potential of plasma CC16 as an early biomarker of lung function impairment in coal miners, mediating analysis was performed to investigate the role of CC16 in the association between dust exposure and lung function decline using the ‘mediation’ package. Cumulative respiratory dust exposure was the predictor (X), plasma CC16 levels were the mediator (M), and lung function was the outcome (Y). Whether baseline plasma CC16 levels could predict future changes in lung function was further explored using RCS models. All statistical analyses were performed using R studio (R version 4.2.2) and SAS 9.4 (SAS Institute Inc., Cary, NC, USA). An alpha error of less than 5% was considered statistically significant.

## Results

### Exposure assessment

A total of 3,151 TWA records were collected. We indirectly validated the exposure assessment by analyzing the relationship between CDE and lung indicators. The fitted lines between CDE and lung indicators showed that lung function decreased as CDE increased (Fig. S2). The *P*-values of Pearson correlation coefficients for all lung indicators were both less than 0.05. These results suggested that the exposure assessment was reasonable.

We summarized the dust exposure levels of different job categories among coal miners. Workers were classified as “Production” and “Security” according to their duties, and they were further divided into nine categories according to their types of operation (Table S1). The median cumulative dust exposure and years of exposure to dust were 35.13 mg/m^3^-years and 17 years, respectively. The mean exposure concentrations were highest for the coal mining machine operators and roadheader operator.

### Characteristics of study participants

Table 1 detailed the characteristics of the participants. The median age was 44 years, and 63.72% of the workers were current smokers. There were 535 (36.62%) individuals with ventilatory dysfunction, with 513 of them having restrictive ventilatory dysfunction. Participants with higher CDE levels were older, had longer dust exposure durations, lower education levels, higher pack-years, lower proportions of never drinkers, more regular exercise, higher blood glucose levels, lower plasma CC16 and lung function levels, higher proportions of ventilatory dysfunction. However, no statistical differences were observed in BMI and abnormal spirometric patterns between the two groups.

**Table 1:** Characteristics of participants stratified by median of CDE.

**Variables^a^**	**Total**	**Median of CDE, mg/m^3^-years**		***P* ^b^**

		**<35.13**	**≥35.13**	
**No. participants**	1461	730	731	
**Age, years**	44 (34, 50)	35 (31, 43)	49 (44, 52)	<0.001
**Duration of dust exposure (years)**	17 (11, 24)	12 (8, 15)	23 (18, 30)	<0.001
**BMI, kg/m^2^**	25.42 (23.23, 27.64)	25.40 (23.15, 27.68)	25.43 (23.31, 27.58)	0.953
**Night shift frequency (times/month)**	8 (0, 10)	7 (0, 10)	9 (0, 12)	0.127
**Education, years**				
low (<9)	552 (37.78)	180 (24.65)	372 (50.89)	<0.001
middle (9–12)	519 (35.52)	250 (34.25)	269 (36.80)	
high (>12)	390 (26.70)	300 (41.10)	90 (12.31)	
**Pack-years**	6.9 (0.0, 17.0)	4.3 (0.0, 11.0)	12 (0.0, 26.0)	<0.001
**Smoking**				
Never	418 (28.61)	230 (31.51)	186 (25.44)	<0.001
Present	931 (63.72)	460 (63.01)	473 (64.71)	
Ever	112 (7.67)	40 (5.48)	72 (9.85)	
**Drinking**				
Never	738 (50.51)	396 (54.25)	342 (46.79)	0.008
Present	661 (45.24)	310 (42.47)	351 (48.02)	
Ever	62 (4.25)	24 (3.29)	38 (5.20)	
**Exercise**				
Never	929 (63.59)	457 (62.60)	472 (64.57)	0.005
Occasional	306 (20.94)	175 (23.97)	131 (17.92)	
Regular	226 (15.47)	98 (13.43)	128 (17.51)	
**Blood glucose, mmol/L**	5.1 (4.9, 5.4)	5.0 (4.8, 5.3)	5.2 (5.0, 5.5)	<0.001
**CC16 (ng/mL)**	22.98 (17.33, 30.57)	24.44 (18.86, 32.83)	21.61 (16.13, 28.22)	<0.001
**Lung function**				
FVC (mL)	3710 (3340, 4180)	3930 (3540, 4370)	3520 (3190, 3880)	<0.001
FEV_1_ (mL)	3150 (2820, 3540)	3390 (3020, 3730)	2970 (2670, 3290)	<0.001
ppFVC (%)	83 (76, 90)	83 (77, 91)	82 (75, 89)	0.001
ppFEV_1_ (%)	88 (81, 95)	89 (81, 96)	88 (80, 95)	0.008
FEV_1_/FVC (%)	85 (82, 88)	86 (82, 89)	85 (81, 88)	<0.001
**Ventilation function**				
Dysfuntion	535 (36.62)	230 (31.51)	305 (41.72)	<0.001
Non-dysfunction	926 (63.38)	500 (68.49)	426 (58.28)	
**Patterns of spirometric abnormality**				
Restrictive	513 (95.88)	224 (97.39)	289 (94.75)	0.187
Obstructive	11 (2.01)	4 (1.74)	7 (2.30)	
Mixed obstructive and restrictive	11 (2.01)	2 (0.87)	9 (2.95)	

^a^Data were presented as n (%) or Med (25th, 75th).

^b^*P* values were calculated from Chi-square (χ^2^) test for categorical variables and Kruskal-Wallis H-test for numerical variables.

### Relative important factors to lung function and CC16

**Fig. 1:**
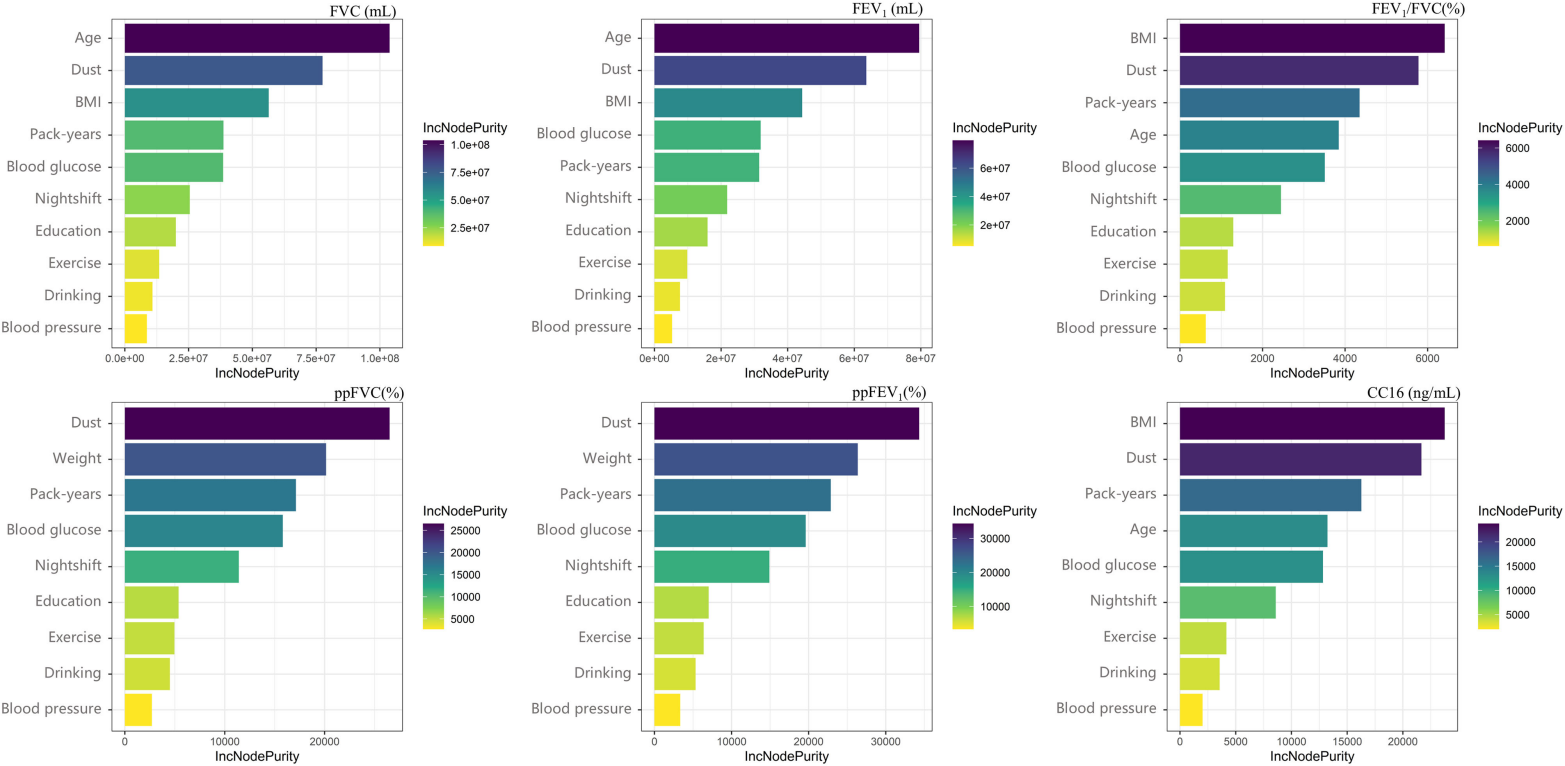
The relative importance of different factors affecting lung function. Different variables are selected as splitting variables for each node in constructing multiple decision trees in RF models. To evaluate the importance of each variable, the average contribution of the variable to the impurity reduction of nodes was calculated when the variable was selected as the splitting variable in different decision trees, which was referred to as the IncNodePurity metric. A higher IncNodePurity value indicates that the split of that node contributes more to the reduction of node impurity, leading to greater improvement in the predictive performance of the model, and consequently, a larger impact on the outcome variable.

We ranked the relative importance of factors affecting lung function and CC16 using RF models. The plot of the relationship between the number of trees and mean squared error (MSE) demonstrated that as the number of trees reached approximately 200, there was no significant change in MSE. Therefore, the number of trees was set as 200 (Fig. S3). The RF models demonstrated that age, CDE, BMI, pack-years, and blood glucose levels were important factors for FVC, FEV_1_, and FEV_1_/FVC and CC16 (Fig. 1). Moreover, CDE, weight, pack-years, and blood glucose levels were important factors for ppFVC and ppFEV_1_. Vegetable and fruit intakes, as well as blood pressure, had a rather low importance on both lung function and CC16 and were excluded from subsequent analyses.

### Dose-effect association of CDE with lung function and CC16

**Fig. 2:**
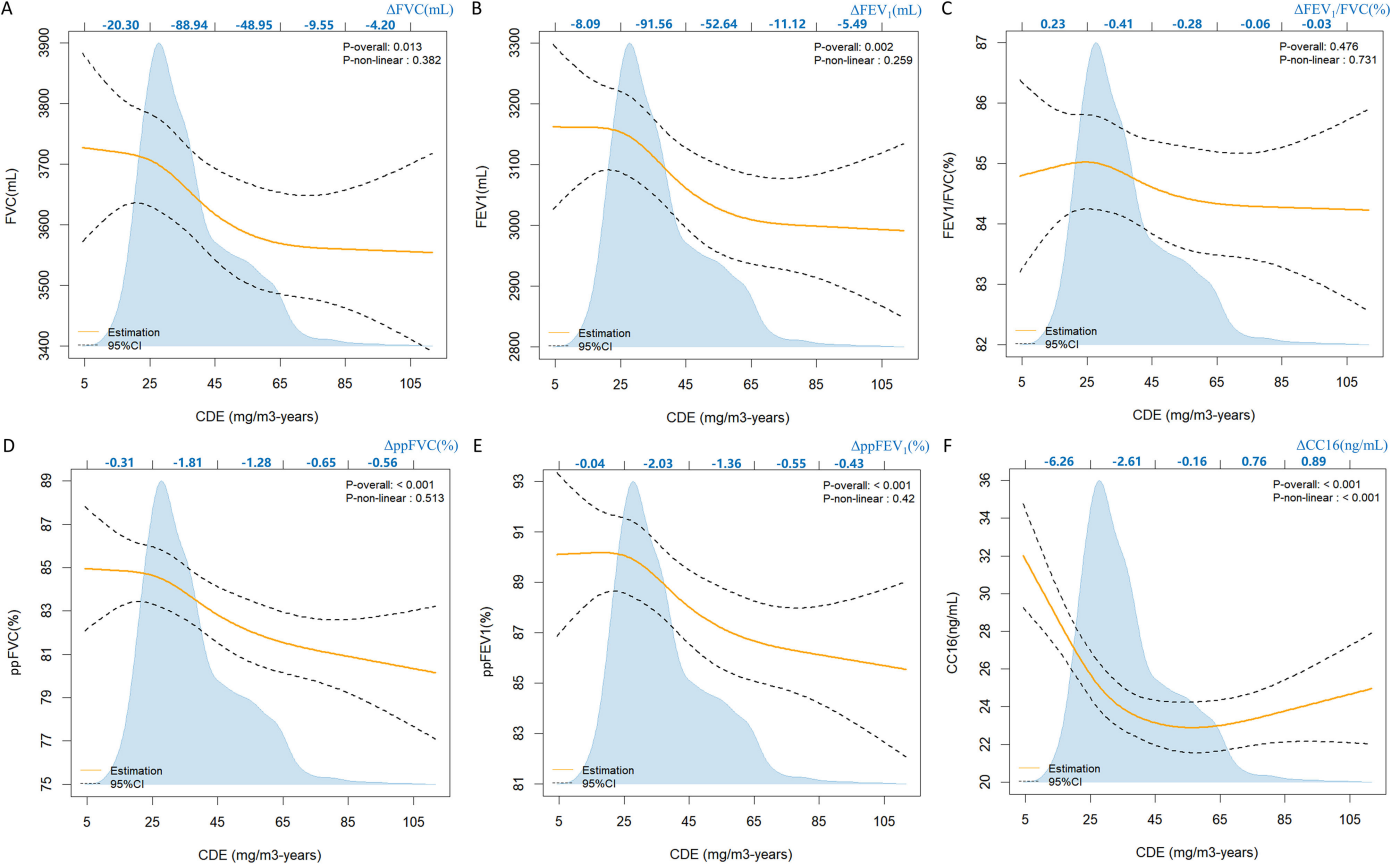
The nonlinear dose-effect associations of CDE with lung function and plasma CC16. The scale on the top coordinate axis is aligned with the x-axis and additionally indicated the changes in lung function and plasma CC16 within each scale interval. The blue background represented the distribution of CDE among workers. Age, BMI, education level, pack-years, drinking status, physical activity level, night shift status and blood glucose were adjusted for FVC, FEV_1_, FEV_1_/FVC. Weight, education level, pack-years, drinking status, physical activity level, night shift status and blood glucose were adjusted for ppFVC and ppFEV_1_. Age, BMI, pack-years, drinking status, physical activity level, night shift status and blood glucose were adjusted for CC16 (ng/mL).

We explored the effects of CDE on lung function using RCS and the results after adjusting for confounding factors (Fig. 2). The CDE for most workers ranged from 5 mg/m^3^-years to 65 mg/m^3^-years. Apart from FEV_1_/FVC, other lung indicators showed significant linear associations with CDE (*P*_overall_ < 0.05; *P*_non-linear_ > 0.05). A rapid decline was observed in FVC, FEV_1_, ppFVC, and ppFEV_1_ when CDE exceeded 25 mg/m^3^-years, particularly within the range of 25 mg/m^3^-years to 65 mg/m^3^-years. Moreover, within this range, the changes in FEV_1_ and ppFEV_1_ were greater than those in FVC and ppFVC (ΔFVC, −137.89 mL; ΔFEV_1_, −144.20 mL; ΔppFVC, −3.09%; ΔppFEV_1_, −3.39%).

There was a significant non-linear association between CC16 levels and CDE (*P*_overall_ < 0.05, *P*_non-linear_ < 0.05). The CC16 levels initially rapidly decreased as CDE increased, then the declining trend slowed and finally transitioned to a gradual upward trend. The CC16 levels continued to decrease when CDE was less than 45 mg/m^3^-years. In particular, the amount of change in CC16 levels between 5 mg/m^3^-years and 25 mg/m^3^-years (−6.26 ng/mL) was greater than that between 25 mg/m^3^-years and 45 mg/m^3^-years (−2.61 ng/mL).

### Effect modification of individual factors on association between dust exposure and lung function

As shown in Fig. 1, in addition to age and dust exposure, smoking status, blood glucose, and BMI had the greatest impacts on lung function. We stratified all participants according to three individual factors and assessed the association between dust exposure and lung function and CC16. Specifically, compared with light smokers (pack-years <6.9), heavy smokers (pack-years ≥6.9) experienced a faster decline in lung function (FEV_1_, ppFVC, ppFEV_1_) due to dust exposure (*P* < 0.05). Smoking modified the association of dust exposure with lung function and this change was more pronounced in ppFEV_1_ (β: −3.5 vs. −6.5) compared with ppFVC (β: −4.3 vs. −5.2). In addition, workers with abnormal blood glucose exhibited faster declines in FVC, FEV_1_, ppFVC, and ppFEV_1_. Overweight or obese workers exhibited faster declines in FVC and FEV_1_ (*P* < 0.05) (Fig. S4A–E).

We categorize workers into two groups based on the median of CDE since CC16 exhibits completely different trends under low-level dust exposure compared to high-level dust exposure. Figure S4F showed the plasma CC16 levels of workers in each subgroup. After considering the influence of other factors, analysis of covariance indicated that heavy smokers exhibited significantly lower plasma CC16 levels than light smokers at the same level of dust exposure. Similarly, overweight or obese workers had lower plasma CC16 levels compared with those with normal BMI. However, no significant difference was observed in plasma CC16 levels among workers with different blood glucose levels.

### Role of CC16 in the associations between dust exposure and lung function

Table 2 showed the mediating effects of CC16 levels on the association between dust exposure and lung function. The results indicated that plasma CC16 levels mediated 13.1% of the proportion between dust exposure and FVC, 15.3% of the proportion between dust exposure and FEV_1_, and 11.1% of the proportion between dust exposure and ppFEV_1_ among all participants. The mediating effects on FEV_1_ (26.0%) and ppFEV_1_ (18.1%) were greater in heavy smokers compared to the overall. In addition, mediating effects were observed only in workers with normal FPG and not in those with abnormal FPG or overweight or obese workers after stratifying the participants based on FPG and BMI.

**Table 2:** Mediation effects of CC16 on associations between dust exposure and lung function among different participants.

**Lung function^a^**	**Variable**	**Total effect β** **(95% CI)**	**Direct effect β** **(95% CI)**	**Mediating effect β** **(95% CI)**	**Proportion mediated (%)**
FVC (mL)	All participants	−184.8 (−322.2, −47.5)	−160.6 (−299.4, −21.8)	−24.2 (−47.1, −1.4)	13.1
	Smoking				
	pack-years <6.9	−179.0 (−383.2, 25.3)	−167.1 (−374.3, 40.0)	−11.8 (47.4, 23.7)	-^b^
	pack-years ≥6.9	−161.2 (−346.0, 25.6)	−109.2 (−295.1, 76.6)	−52.0 (−89.8, −14.1)	-^b^
	Blood glucose				
	<6.1	−160.1 (−302.8, −17.5)	−133.0 (−277.2, 11.1)	−27.1 (−51.3, −2.9)	16.9
	≥6.1	−599.2 (−1097.9, −100.5)	−609.6 (−1118.3, −100.9)	10.4 (−91.0, 111.8)	-^b^
	BMI				
	<24	8.6 (−209.7, 227.0)	35.0 (−184.8, 254.9)	−26.4 (−61.2, 8.4)	-^b^
	≥24	−300.0 (−474.7, −125.2)	−274.3 (−451.6, −97.0)	−25.7 (−58.2, 6.8)	-^b^

FEV_1_ (mL)	All participants	−186.7 (−306.7, −66.6)	−158.2 (−279.4, −37.0)	−28.5 (−49.4, −7.6)	15.3
	Smoking				
	pack-years <6.9	−125.6 (−303.0, 51.9)	−111.3 (−291.2, 68.7)	−14.3 (−45.5, 16.9)	-^b^
	pack-years ≥6.9	−219.4 (−382.1, −56.7)	−163.5 (−326.5, −0.5)	−56.0 (−92.1, −19.8)	26.0
	Blood glucose				
	<6.1	−161.5 (−285.9, −37.0)	−130.3 (−255.8, −4.7)	−31.2 (−53.4, −9.0)	19.3
	≥6.1	−598.7 (−1043.0, −154.4)	−606.7 (−1059.9, −153.4)	8.0 (−82.3, 98.2)	-^b^
	BMI				
	<24	−44.4 (−234.0, 145.23)	−14.3 (−204.9, 176.2)	−30.1 (−62.6, 2.4)	-^b^
	≥24	−273.8 (−426.9, −120.8)	−244.8 (−399.9, −89.6)	−29.1 (−58.3, 0.1)	-^b^

ppFVC (%)	All participants	−4.8 (−7.1, −2.6)	−4.6 (−6.9, −2.3)	−0.2 (−0.7, 0.2)	-^b^
	Smoking				
	pack-years <6.9	−4.3 (−7.3, −1.3)	−4.3 (−7.3, −1.2)	0.0 (−0.6, 0.6)	-^b^
	pack-years ≥6.9	−5.3 (−8.6, −2.0)	−4.6 (−7.9, −1.3)	−0.7 (−1.4, −0.0)	13.2
	Blood glucose				
	<6.1	−4.6 (−6.9, −2.3)	−4.3 (−6.6, −1.9)	−0.3 (−0.8, 0.1)	-^b^
	≥6.1	−12.0 (−20.8, −3.2)	−13.2 (−22.0, −4.3)	1.2 (−1.0, 3.4)	-^b^

ppFEV_1_ (%)	All participants	−4.9 (−7.6, −2.5)	−4.5 (−7.1, −1.9)	−0.6 (−1.1, −0.1)	11.1
	Smoking				
	pack-years <6.9	−3.6 (−6.9, −0.2)	−3.4 (−6.8, 0.0)	−0.2 (−0.9, 0.5)	-^b^
	pack-years ≥6.9	−6.7 (−10.4, −2.9)	−5.5 (−9.2, −1.7)	−1.2 (−2.0, −0.3)	18.1
	Blood glucose				
	<6.1	−4.8 (−7.4, −2.2)	−4.1 (−6.8, −1.5)	−0.7 (−1.2, −0.1)	13.9
	≥6.1	−13.0 (−23.6, −2.4)	−14.2 (−24.9, −3.5)	1.2 (−1.3, 3.7)	-^b^

FEV_1_/FVC (%)	All participants	−0.7 (−2.2, 0.7)	−0.5 (−2.0, 0.9)	−0.2 (−0.4, 0.0)	-^b^
	Smoking				
	pack-years <6.9	0.5 (−1.4, 2.4)	0.7 (−1.2, 2.6)	−0.1 (−0.5, 0.2)	-^b^
	pack-years ≥6.9	−1.9 (−4.0, 0.2)	−1.6 (−3.7, 0.5)	−0.3 (−0.6, 0.1)	-^b^
	Blood glucose				
	<6.1	−0.6 (−2.0, 0.9)	−0.4 (−1.8, 1.1)	−0.2 (−0.5, 0.0)	-^b^
	≥6.1	−3.2 (−8.3, 2.0)	−3.2 (−8.5, 2.0)	0.1 (−1.0, 1.1)	-^b^
	BMI				
	<24	−1.4 (−3.8, 1.0)	−1.2 (−3.6, 1.2)	−0.2 (−0.5, 0.2)	-^b^
	≥24	−0.4 (−2.2, 1.3)	−0.2 (−2.0, 1.5)	−0.2 (−0.5, 0.1)	-^b^

^a^Age, BMI, education level, pack-years, drinking status, physical activity level, night shift status and blood glucose were adjusted for FVC, FEV_1_, FEV_1_/FVC. Weight, education level, pack-years, drinking status, physical activity level, night shift status and blood glucose were adjusted for ppFVC and ppFEV_1_.

^b^Proportion mediated could not be tested because the total effect or direct effect were not significant.

### Low baseline CC16 levels predict poor future lung function

**Fig. 3:**
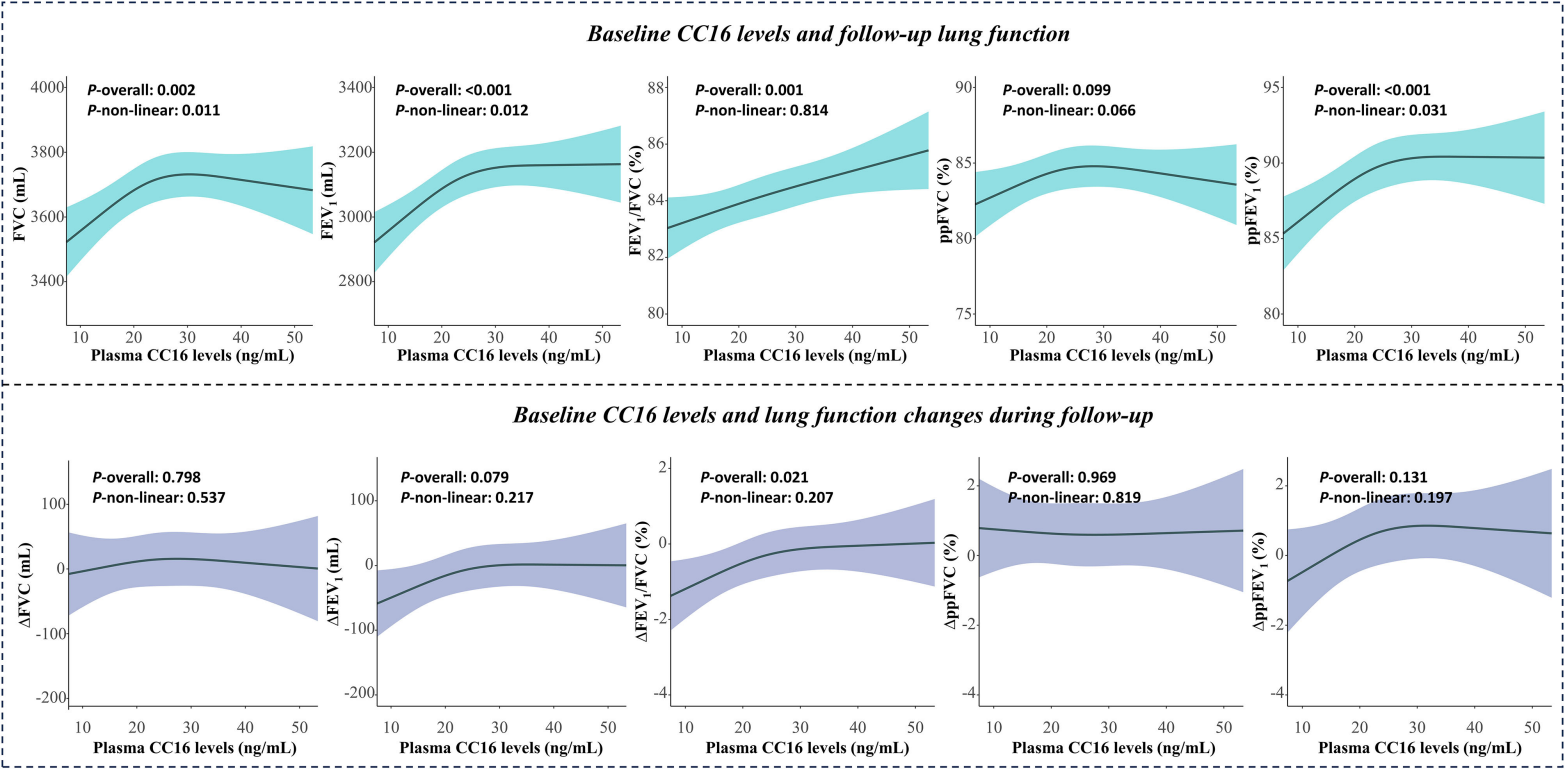
Restricted cubic spline analysis of the nonlinear associations of CDE with lung function at follow-up. Age, BMI, education level, pack-years, drinking status, physical activity level, night shift status and blood glucose were adjusted for FVC, FEV_1_, FEV_1_/FVC, ΔFVC, ΔFEV_1_ and ΔFEV_1_/FVC. Weight, education level, pack-years, drinking status, physical activity level, night shift status and blood glucose were adjusted for ppFVC, ppFEV_1_, ΔppFVC, and ΔppFEV_1_.

The basic characteristics of workers at baseline and follow-up did not show significant differences (Table S2). Results from RCS models showed that baseline CC16 levels were nonlinearly dose-effect associated with follow-up FVC, FEV_1_, and ppFEV_1_, and linearly dose-effect associated with FEV_1_/FVC (Fig. 3). Follow-up FEV_1_ and ppFEV_1_ initially increased and then plateaued as CC16 levels increased, while FEV_1_/FVC remained elevated. Baseline CC16 levels were significantly associated with the change in FEV_1_ and FEV_1_/FVC, and the reduction in lung function was more pronounced with low baseline CC16 levels. In addition, baseline CC16 levels showed a marginally significant association with the change in ppFEV_1_ (*P* = 0.079).

## Discussion

This study quantitatively assessed the dust exposure levels of 1,461 coal miners by retrospectively combining occupational history and 3,151 historical monitoring records, and investigated the associations between CDE and lung function as well as plasma CC16 levels. The potential of CC16 to reflect lung injury was confirmed from multiple perspectives. The exploration demonstrated a rapid decline in lung indicators when CDE was above 25 mg/m^3^-years. Plasma CC16 levels consistently and rapidly decreased as dust exposure levels increased when the CDE was less than 45 mg/m^3^-years. These results indicated that plasma CC16 levels was more sensitive than lung indicators at low cumulative dust exposure levels. Further analysis revealed that dust exposure significantly impacted lung function through CC16. More importantly, we found plasma CC16 levels could reflect future lung function impairment through follow-up.

The representation of dust exposure in terms of years of service was imprecise because of substantial variations in dust concentrations across different occupations. To overcome this limitation, we conducted a quantitative assessment of the workers’ dust exposure levels. The coal mining machine driver and roadheader operator were exposed to higher dust concentrations, which is consistent with the findings of Pandey [21]. This is because the cutting of coal and rock during mining operations generated a substantial amount of respirable dust, causing the coal mining face and heading face to became the primary sources of dust in underground coal mining [22, 23].

According to previous research, achieving an early diagnosis of CWP is challenging during the most effective preventive stage, as no evident symptoms or notable changes in lung function are evident during the initial phases of coal dust exposure [24]. The current study validated these findings. A decline in lung indicators was not apparent when the CDE remained <25 mg/m^3^-years. This phenomenon may be attributed to the robust compensatory mechanisms of the lungs. However, there was a rapid deterioration in lung function once CDE surpassed this threshold. The permissible concentration-time weighted average (PC-TWA) for respirable coal dust was set at 2.5 mg/m^3^ in accordance with the Chinese national standard [25]. This indicated that no obvious decline in lung indicators was observed during the continuous 10-year work period for coal miners exposed to PC-TWA. The ventilation dysfunction type associated with CWP was mainly restrictive ventilation dysfunction (indicated by ppFVC) in the early stage, and obstruction (indicated by FEV_1_/FVC) was most common in the middle and late stages, followed by mixed ventilation dysfunction. In our study, a greater number of workers had restrictive ventilatory dysfunction, indicating that the participants were in the early stages of ventilatory dysfunction. An overall downward trend was observed in ppFVC with increasing exposure levels, suggesting that continued exposure to coal dust may result in restrictive ventilation dysfunction. However, we only observed a significant difference in FEV_1_/FVC between the high and low dust exposure groups, and did not observe a significant association between CDE and FEV_1_/FVC suggesting that the participants had only mild airflow obstruction. In addition, impaired pulmonary ventilation can be reflected by ppFEV_1_ [14]. An overall downward trend was observed in FEV_1_ (%) with increasing dust exposure levels, indicating increased severity of impaired lung ventilation.

This study found overweight or obese workers experienced a higher rate of decline in FVC than that of normal weight worker at any level of dust exposure. The negative impact of smoking on lung function has long been well-established [26], and previous studies have shown that smoking has a greater effect on FEV_1_ [27]. Our results confirmed that heavy smokers exhibited a greater rate of decline in ppFEV_1_ than in ppFVC. Additionally, FEV_1_ and ppFEV_1_ decreased faster in heavy smoking than in light smoking. Furthermore, workers with abnormal blood glucose levels experienced a higher rate of decline in FVC and ppFVC compared with that in workers with normal blood glucose levels. These results found the vulnerable group of coal miners and emphasized the importance of maintaining weight and blood glucose levels, as well as reducing smoking.

In general, acute exposures to factors that lead to epithelial injury result in a transient increase in circulating CC16 due to increased lung permeability [28]. But both humans and animals tend to exhibit decreased plasma CC16 levels and bronchoalveolar lavage fluid after repeated environmental exposure [5]. Clinical studies have demonstrated that serum CC16 levels are decreased in patients with asthma and chronic obstructive pulmonary disease (COPD) [29, 30]. CC16 deficient mice exhibit heightened lung epithelial injury, increased airway inflammation, and enhanced vulnerability to oxidative stress and infectious agents [31]. We found the CC16 levels were more sensitive at low dust exposure levels compared with lung indicators. The rapid decline in CC16 levels could be attributed to chronic dust exposure, which damaged lung epithelial cells and/or CC16 gene downregulation, and impaired the production and release of CC16. Furthermore, the presence of inflammatory mediators and oxidative stress associated with dust exposure may contribute to decreased CC16 levels by altering alveolar epithelial permeability [32]. Subsequently, plasma CC16 levels entered a plateau phase when exposed to high dust levels, which may indicate that club cells were severely damaged and a new equilibrium was reached to maintain a certain level of CC16 in the presence of ongoing dust-induced damage and inflammation. Previous studies have shown that overweight or obese workers and smokers have low CC16 levels [33, 34]. Similar results were obtained in the present study and no differences in plasma CC16 levels among workers with different blood glucose levels, which was consistent with an earlier study [35].

A clinical study on patients with COPD found a positive association between CC16 levels and FEV_1_ [10]. A recent study showed that low CC16 mRNA expression levels in bronchial epithelial cells are associated with asthma severity [36]. These studies highlighted the association between CC16 and changes in lung function, but the impact of coal dust exposure on lung function decline, particularly within populations with varying characteristics, had been insufficiently explored. This study demonstrated that coal miners exposed to dust experienced partial impacts on lung function through the reduction in CC16 levels. The mediating role of CC16 was more pronounced in individuals with a heavy smoking history, which suggested that under the influence of smoking, CC16 had played a more pivotal role in the coal dust-induced deterioration of lung function. It was further elucidated that Low baseline CC16 levels predict poor future lung function through follow-up studies of coal miners. These results illustrated the importance of monitoring changes in CC16 levels. Overall, this study found significant potential for CC16 levels in reflecting early lung function impairment in coal miners, making it a promising biomarker for monitoring lung function alterations in this population.

This study had several advantages. First, the exposure levels of each worker were assessed quantitatively, which increased the accuracy of our findings. Second, important individual factors that affect lung function and CC16 were identified using machine learning, which provided a basis for analyses within different groups and the identification of vulnerable groups. Additionally, this study explores the potential of CC16 to reflect early lung injury in coal miners from multiple perspectives, providing evidence for validating the use of plasma CC16 as a new indicator for monitoring lung function impairment in coal miners.

The present study had several limitations. First, the assessment of exposure levels in coal mine workers was conducted longitudinally, but the examination of plasma CC16 levels was limited to a single time point, which impeded the observation of dynamic changes in plasma CC16 levels. Non-occupational lung diseases may also influence CC16 levels. Therefore, abnormal CC16 levels may lead to a certain false-positive rate among coal miners. Although an acceptable level of false positives among coal miners may help in early identification of high-risk individuals and strengthen health monitoring, whether CC16 can serve as a reliable biomarker still requires further validation. And the study did not include data on the general population or individuals with CWP, thus precluding the acquisition of baseline CC16 levels among the normal population or plasma CC16 levels in patients at different stages of CWP. While CDE reflects respirable dust mass, variations in particle size and shape across job types were not assessed. Future studies should integrate physicochemical dust characteristics to refine exposure assessments and further explore its health impacts. Additionally, the mechanism by which CC16 levels maintain balance or undergo slow recovery in the context of high-level dust exposure is currently unclear due to limitations in epidemiological research. Future studies will require larger sample sizes and longer follow-up periods to validate the effectiveness of CDE and assess the potential of CC16 as a biomarker for coal miners.

## Conclusions

Our findings provide relevant evidence for the association between dust exposure, reduced plasma CC16 levels and reduced lung function in coal miners. CC16 mediates the effect of dust exposure on lung function decline and proves to be more sensitive than lung indicators in reflecting early lung injury among coal mine workers. Monitoring CC16 levels holds potential for tracking lung injury in coal miners.

## Abbreviations

CC16: club cell secretory protein
CDE: cumulative respiratory dust exposure
RCS: restricted cubic spline
FVC: forced vital capacity
FEV_1_: forced expiratory volume in the first second
CWP: coal workers’ pneumoconiosis
TWA: time-weighted average concentration
ATS: American Thoracic Society
ERS: European Respiratory Society
ppFVC: the percentage of predicted forced vital capacity
ppFEV_1_: the percentage of predicted forced expiratory volume in one second
FPG: fasting plasma glucose
SBP: systolic blood pressure
DBP: diastolic blood pressure
PC-TWA: permissible concentration-time weighted average
COPD: chronic obstructive pulmonary disease

## Supplementary information

**Additional file 1:** **Table S1** Dust exposure levels of different types of work. **Table S2** Basic characteristics of follow-up participants and baseline participants. **Figure S1** Coal miner recruitment flowchart. **Figure S2** The fitting lines between cumulative dust exposure and lung function indicators. **Figure S3** Tuning of the number of trees in the random forest regression model. **Figure S4** Dose-effect associations of CDE with lung function among all participants and different subgroups.

## Declarations

### Availability of data and materials

The data that support the findings of this study are available from the corresponding author (J.Y.) upon reasonable request and with permission of the Shanxi Medical University, Shanxi, China.
