# Supplementary material for: Plasma club cell secretory protein reflects early lung injury: comprehensive epidemiological evidence
Source: Environ Health Prev Med. 2025 Apr 15;30:26. doi: 10.1265/ehpm.24-00335 (PMC12006028; doi:10.1265/ehpm.24-00335)
Supplement: Supplementary file 1 — Additional file 1: Table S1 Dust exposure levels of different types of work. Table S2 Basic characteristics of follow-up participants and baseline participants. Figure S1 Coal miner recruitment flowchart. Figure S2 The fitting lines between cumulative dust exposure and lung function indicators. Figure S3 Tuning of the number of trees in the random forest regression model. Figure S4 Dose-effect associations of CDE with lung function among all participants and different subgroups. [file ehpm-30-026-s001.pdf]

Table S1 Dust exposure levels of different types of work.

Duty	Type of operation	Type of work	Number	CDE (mg/m^3 - years)[a]	Working years of exposed to dust (year)[a]	The mean exposure concentration (mg/m^3)
	All participants	——	1461	35.13 (1.92, 146.49)	17 (1, 40)	2.07
Production	mining operation	roof bolter	194	30.31 (2.08, 88.34)	15 (1, 33)	2.02
		coal miner	24	25.84 (11.47, 65.53)	17 (7, 33)	1.52
		drivage worker	63	33.50 (9.77, 74.44)	16 (5, 27)	2.09
		mine maintenance electrician	70	35.14 (2.76, 80.76)	18 (1, 33)	1.85
		hydraulic support worker	43	40.80 (15.43, 88.96)	17 (6, 35)	2.40
		the coal mining machine driver	34	64.18 (18.06, 146.49)	15 (3, 32)	4.28
		underground general worker	61	25.79 (2.71, 86.00)	15 (2, 40)	1.72
		hydraulic pump worker	9	22.06 (15.41, 23.66)	17 (14, 19)	1.30
		roadheader operator	9	66.24 (43.11, 80.68)	15 (8, 17)	4.42
	underground blasting operation	underground blaster	19	26.22 (5.75, 53.76)	17 (4, 19)	1.54
Security	underground electrical work	substation electrician	4	25.02 (19.15, 36.58)	18 (13, 36)	1.39
		mining electrical fitter	12	33.01 (18.82, 69.61)	17 (8, 32)	1.94
		winder operator	14	39.36 (3.62, 84.19)	17 (4, 32)	2.32
		mine electrical installer	12	25.07 (9.5, 36.03)	25 (8, 36)	1.00
		mine mechanical installer	25	32.90 (15.65, 74.69)	18 (7, 33)	1.83
	safety monitoring work	safety instrument monitoring worker	9	57.69 (21.96, 73.36)	31 (10, 39)	1.84
	gas inspection operation	gas inspection worker	46	27.76 (8.22, 74.28)	17 (6, 38)	1.63

safety inspection operation	safety inspector	9	30.86 (14.03, 75.22)	16 (5, 31)	1.93
	pipeline worker	21	23.34 (11.45, 51.52)	10 (5, 26)	2.33
	mine ventilation worker	11	27.25 (7.13, 71.73)	13 (3, 35)	2.10
	rock bolt and shotcrete worker	6	20.23 (1.92, 33.68)	14 (1, 19)	1.45
	tunnel excavation and masonry worker	17	38.84 (15.01, 64.35)	18 (7, 33)	2.16
transportation operation	belt operator	61	38.18 (6.76, 117.5)	17 (3, 36)	2.25
	electric locomotive operator	40	64.04 (16.29, 103.35)	25 (7, 38)	2.56
	mine track worker	15	51.52 (14.82, 127.55)	26 (5, 37)	1.98
	underground haulage worker	23	68.35 (4.03, 131.49)	26 (2, 38)	2.63
	signalman	2	28.83[b]	15[b]	1.92
	hoist operator	1	11.11[b]	14[b]	0.79
gas drainage operation	mine ventilation worker	2	49.08[b]	24[b]	2.05
	gas drainage worker	17	14.8 (6.4, 22.65)	8 (4, 18)	1.85
	main fan operator	10	31.21 (26.61, 85.72)	14 (10, 38)	2.23
mine surveying operation	drill man	20	40.01 (13.74, 87.31)	16 (6, 33)	2.56
	mine pump worker	25	21.37 (4.03, 80.76)	11 (2, 33)	1.94
	mine surveyor	1	65.57[b]	32[b]	2.05
—— ——	workers with multiple job histories	532	39.21 (2.19, 133.93)	19 (1, 40)	2.06

[a] Data were presented as Med (Minimum, Maximum).

[b] Data were presented as the original value of one person or the mean of two people.

Table S2 Basic characteristics of follow-up participants and baseline participants

Variables[a]	Total	Follow up	*P*[b]
**No. participants**	1461	1268	
**Age, years**	44 (34, 50)	44 (34, 50)	0.917
**BMI, kg/m^2**	25.42 (23.23, 27.64)	25.39 (23.18, 27.62)	0.841
**Night shift frequency（times/month）**	8 (0, 10)	8 (0, 12)	0.947
**Education, years**			
low (<9)	552 (37.78)	468 (36.91)	0.874
middle (9-12)	519 (35.52)	461 (36.36)	
high (>12)	390 (26.70)	339 (26.73)	
**Pack-years**	6.9 (0.0, 17.0)	7.2 (0.0, 17.0)	0.848
**Drinking**			
Never	738 (50.51)	645 (50.87)	0.981
Present	661 (45.24)	569 (44.87)	
Ever	62 (4.25)	54 (4.26)	
**Exercise**			
Never	929 (63.59)	821 (64.75)	0.701
Occasional	306 (20.94)	265 (20.90)	
Regular	226 (15.47)	182 (14.35)	
**Blood glucose, mmol/L**	5.1 (4.9, 5.4)	5.1 (4.9, 5.4)	0.684
**CC16 (ng/mL)**	22.98 (17.33, 30.57)	23.07 (17.33, 30.66)	0.919
**Lung function**			
FVC(mL)	3710 (3340, 4180)	3720 (3350, 4180)	0.625
FEV_1(mL)	3150 (2820, 3540)	3160 (2825, 3550)	0.732
ppFVC(%)	83 (76, 90)	83 (76, 90)	0.646
ppFEV_1(%)	88 (81, 95)	88 (80, 95)	0.837
FEV_1/FVC(%)	85 (82, 88)	85 (82, 88)	0.755

[a] Data were presented as n (%) or Med (25th, 75th).

[b] *P* values were calculated from Chi-square (χ^2) test for categorical variables and Kruskal-Wallis H-test for numerical variables.

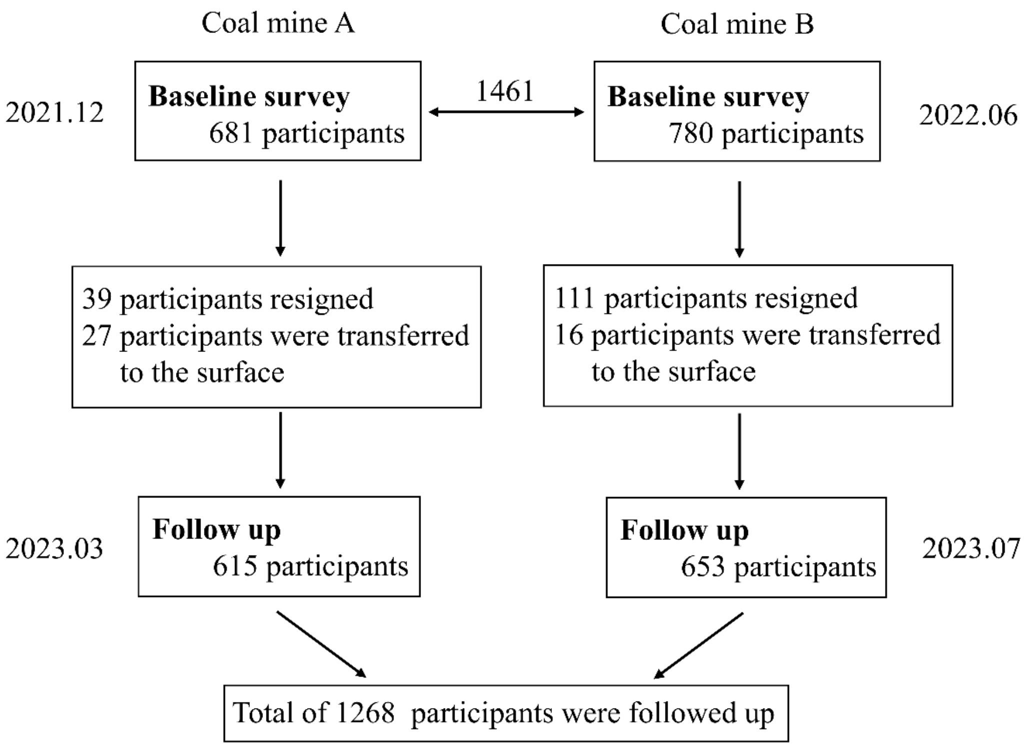

Figure S1 Coal miner recruitment flowchart.

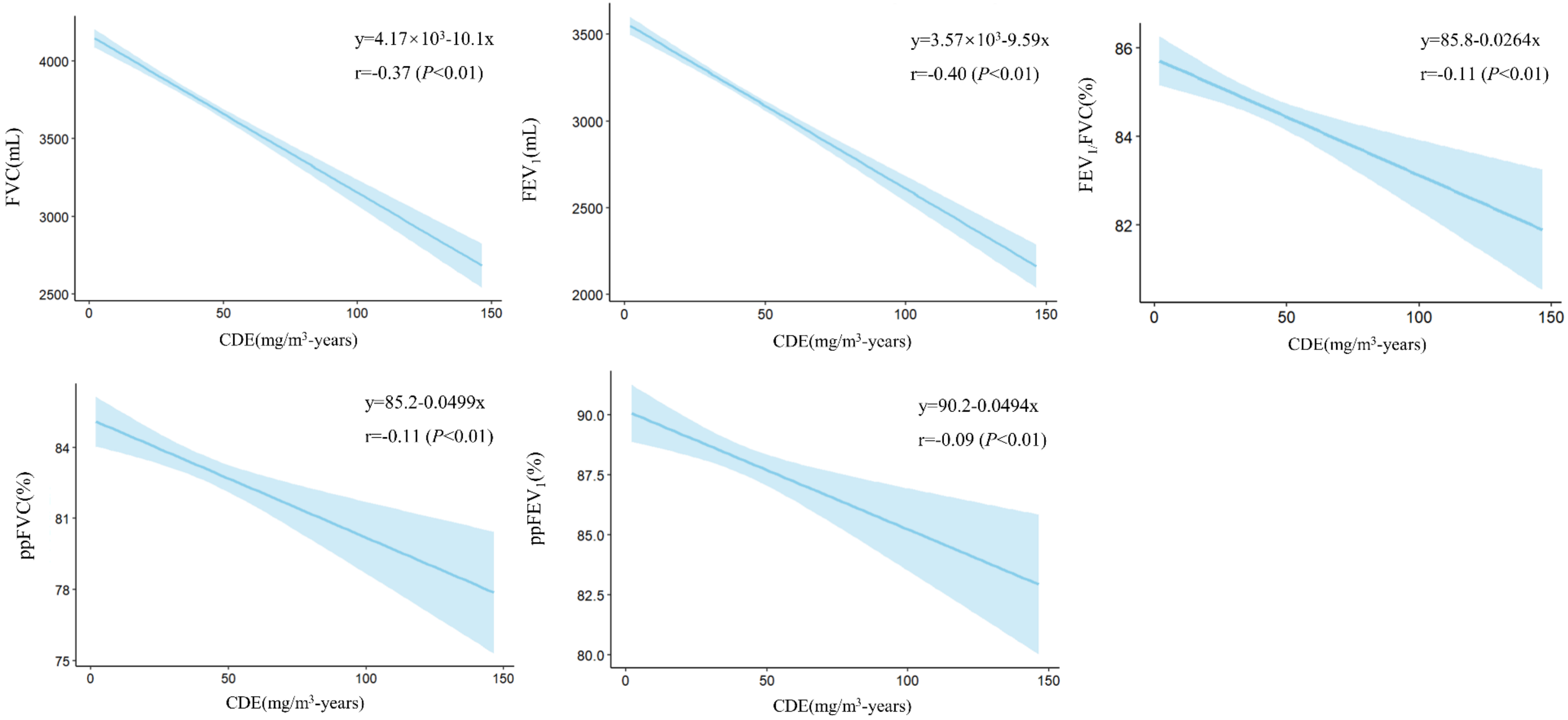

Figure S2 The fitting lines between cumulative dust exposure and lung function indicators. The blue shading represents the 95% CI.

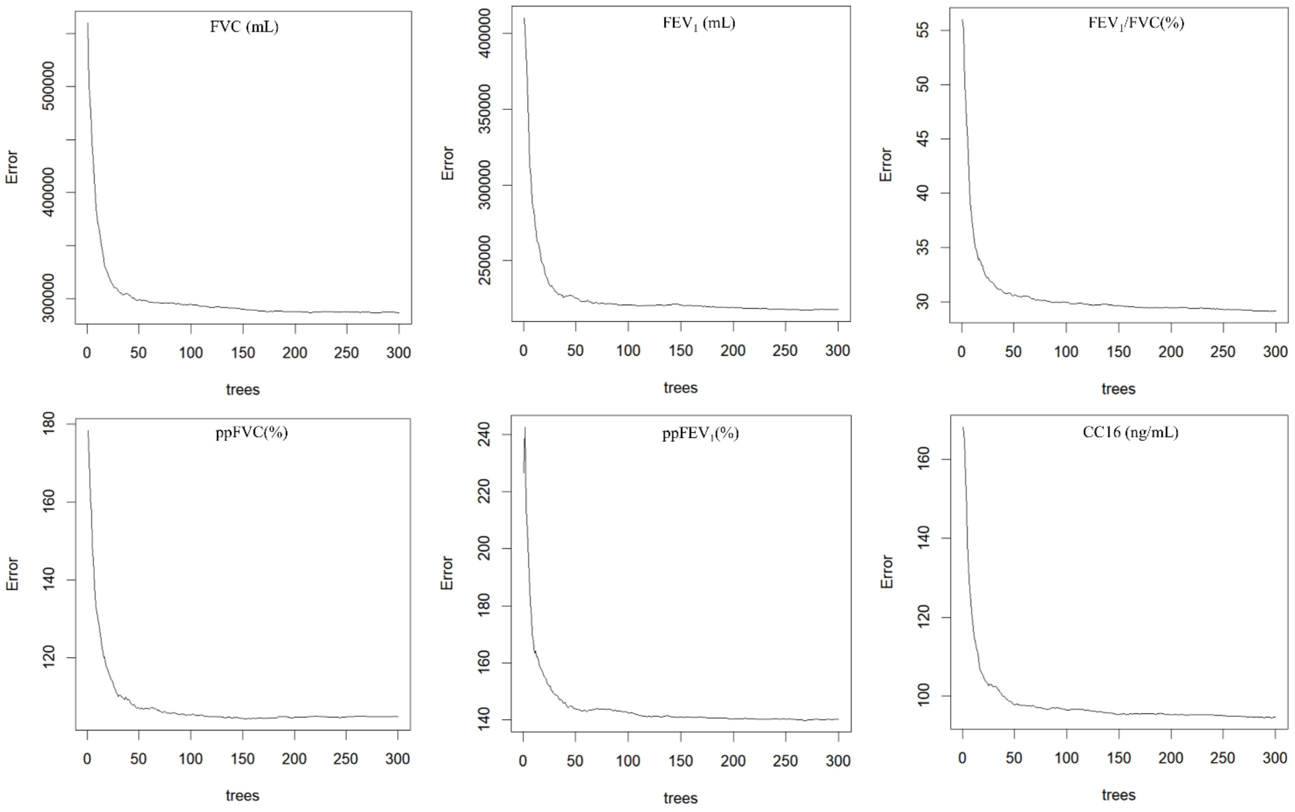

Figure S3 Tuning of the number of trees in the random forest regression model. The performance of RF models was improved by adjusted number of trees. A smaller mean squared error (MSE) indicated better model performance.

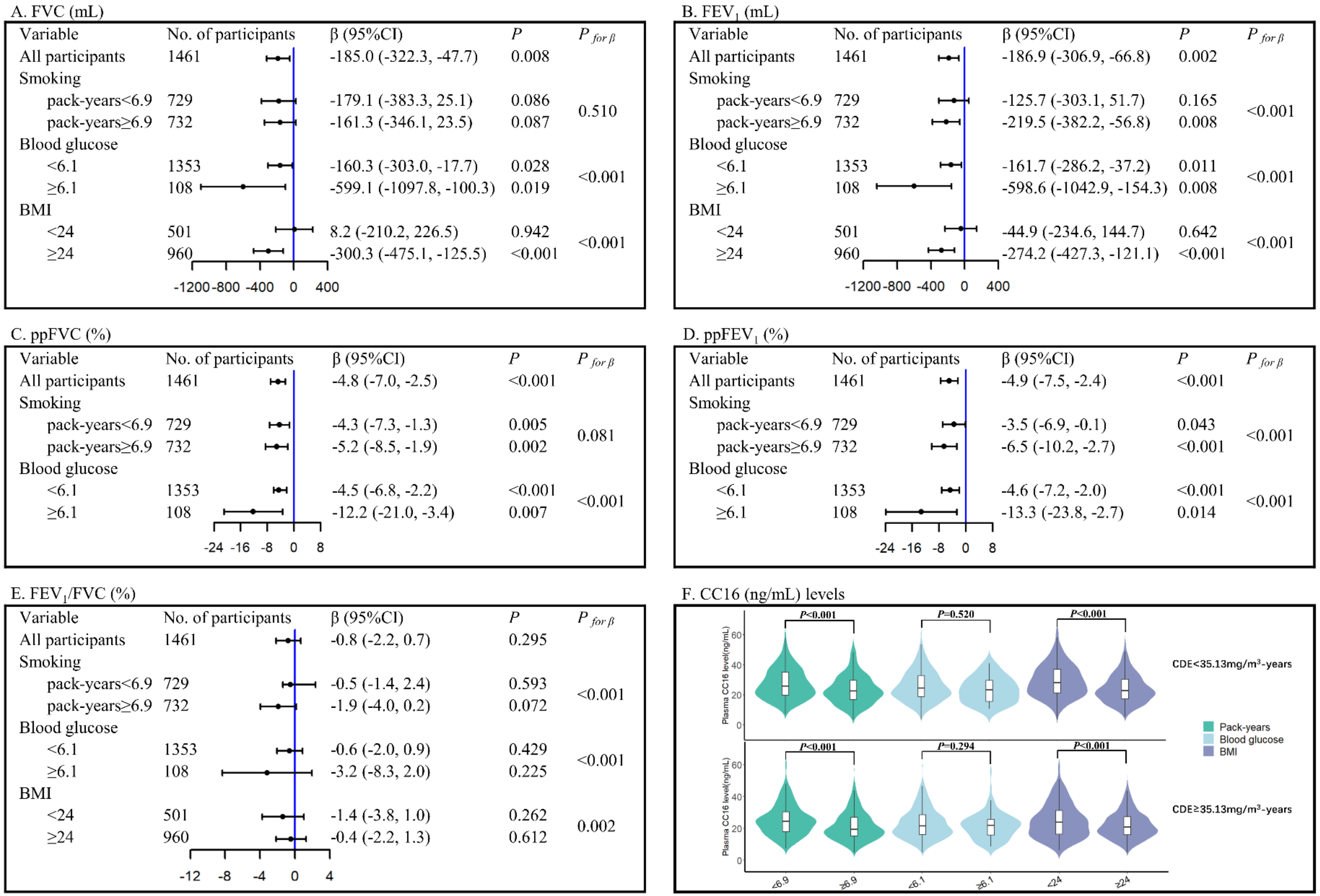
A. FVC (mL)
Variable | No. of participants | β (95%CI) | P | P for β
All participants | 1461 | -185.0 (-322.3, -47.7) | 0.008
Smoking
pack-years<6.9 | 729 | -179.1 (-383.3, 25.1) | 0.086 | 0.510
pack-years≥6.9 | 732 | -161.3 (-346.1, 23.5) | 0.087
Blood glucose
<6.1 | 1353 | -160.3 (-303.0, -17.7) | 0.028 | <0.001
≥6.1 | 108 | -599.1 (-1097.8, -100.3) | 0.019
BMI
<24 | 501 | 8.2 (-210.2, 226.5) | 0.942 | <0.001
≥24 | 960 | -300.3 (-475.1, -125.5) | <0.001
-1200 -800 -400 0 400
B. FEV1 (mL)
Variable | No. of participants | β (95%CI) | P | P for β
All participants | 1461 | -186.9 (-306.9, -66.8) | 0.002
Smoking
pack-years<6.9 | 729 | -125.7 (-303.1, 51.7) | 0.165 | <0.001
pack-years≥6.9 | 732 | -219.5 (-382.2, -56.8) | 0.008
Blood glucose
<6.1 | 1353 | -161.7 (-286.2, -37.2) | 0.011 | <0.001
≥6.1 | 108 | -598.6 (-1042.9, -154.3) | 0.008
BMI
<24 | 501 | -44.9 (-234.6, 144.7) | 0.642 | <0.001
≥24 | 960 | -274.2 (-427.3, -121.1) | <0.001
-1200 -800 -400 0 400
C. ppFVC (%)
Variable | No. of participants | β (95%CI) | P | P for β
All participants | 1461 | -4.8 (-7.0, -2.5) | <0.001
Smoking
pack-years<6.9 | 729 | -4.3 (-7.3, -1.3) | 0.005 | 0.081
pack-years≥6.9 | 732 | -5.2 (-8.5, -1.9) | 0.002
Blood glucose
<6.1 | 1353 | -4.5 (-6.8, -2.2) | <0.001 | <0.001
≥6.1 | 108 | -12.2 (-21.0, -3.4) | 0.007
-24 -16 -8 0 8
D. ppFEV1 (%)
Variable | No. of participants | β (95%CI) | P | P for β
All participants | 1461 | -4.9 (-7.5, -2.4) | <0.001
Smoking
pack-years<6.9 | 729 | -3.5 (-6.9, -0.1) | 0.043 | <0.001
pack-years≥6.9 | 732 | -6.5 (-10.2, -2.7) | <0.001
Blood glucose
<6.1 | 1353 | -4.6 (-7.2, -2.0) | <0.001 | <0.001
≥6.1 | 108 | -13.3 (-23.8, -2.7) | 0.014
-24 -16 -8 0 8
E. FEV1/FVC (%)
Variable | No. of participants | β (95%CI) | P | P for β
All participants | 1461 | -0.8 (-2.2, 0.7) | 0.295
Smoking
pack-years<6.9 | 729 | -0.5 (-1.4, 2.4) | 0.593 | <0.001
pack-years≥6.9 | 732 | -1.9 (-4.0, 0.2) | 0.072
Blood glucose
<6.1 | 1353 | -0.6 (-2.0, 0.9) | 0.429 | <0.001
≥6.1 | 108 | -3.2 (-8.3, 2.0) | 0.225
BMI
<24 | 501 | -1.4 (-3.8, 1.0) | 0.262 | 0.002
≥24 | 960 | -0.4 (-2.2, 1.3) | 0.612
-12 -8 -4 0 4
F. CC16 (ng/mL) levels
P<0.001
P=0.520
P<0.001
Plasma CC16 level(ng/mL)
CDE<35.13mg/m³-years
Pack-years
Blood glucose
BMI
P<0.001
P=0.294
P<0.001
Plasma CC16 level(ng/mL)
CDE≥35.13mg/m³-years
<6.9
≥6.9
<6.1
≥6.1
<24
≥24

Figure S4 Dose-effect associations of CDE with lung function among all participants and different subgroups. Notes: Age, BMI, education level, pack-years, drinking status, physical activity level, night shift status and blood glucose were adjusted for FVC, FEV_1, FEV_1/FVC. Weight, education level, pack-years, drinking status, physical activity level, night shift status and blood glucose were adjusted for ppFVC and $ppFEV_1$. The $P_{for\ \beta}$ reflected the differences in the regression coefficients among different subgroups by calculating the z value. Analysis of covariance was used to compare plasma CC16 levels in different groups.
